# Instructed speed and accuracy affect binding

**DOI:** 10.1007/s00426-024-01927-y

**Published:** 2024-02-09

**Authors:** Silvia Selimi, Birte Moeller

**Affiliations:** Cognitive Psychology, University of Trier, 54286 Trier, Germany

## Abstract

In the past few decades, binding and retrieval mechanisms have gained increased interest in research on human action control. Recent studies show that these mechanisms also play a role in the control of multiple independent actions. Here, two or more successively executed responses seem to be bound to each other so that repeating one of them can retrieve the other, affecting performance in this second response and resulting in so-called response–response binding effects. Binding effects are typically found in the response time data and, somewhat less reliably, also in the error rates. Whether binding effects show in the response times, the error rates, or both, is likely influenced by the current speed–accuracy settings of the participants, with binding effects more likely showing in error rates under a speed setting, while more likely showing in RTs under an accuracy setting. Alternatively, different speed–accuracy settings might also entail changes in executive control, affecting the size of observed binding effects. In this study, we tested these assumptions by comparing binding effects under different speed–accuracy settings that were induced via instructions focusing on speed, accuracy, or both (ambivalent). Binding effects were observed in response times independent of instructions, while in error rates, they only showed under speed or ambivalent instructions. These findings indicate that binding effects can be affected by instructions regarding speed and accuracy.

**Supplementary Information:**

The online version contains supplementary material available at 10.1007/s00426-024-01927-y.

## Introduction

According to current action control theories, when planning and conducting an action, stimulus, response, and effect features belonging to that action are bound into a short-term memory trace called an *event file* (Hommel, 2004). Repeating any of the bound features can then start retrieval of the other features later on, which affects further performance (Frings et al., 2020; Hommel, 1998; Logan, 1988; Schmidt et al., 2016). For example, repeating a feature that was bound to a response will trigger retrieval of the response. If the response is repeated as well, retrieval of the (compatible) response due to feature repetition facilitates responding. In contrast, if the required response changes, the retrieved and required responses are incompatible and retrieval due to feature repetition leads to significantly less facilitation or even impairment. Statistically, this binding effect is indicated by an interaction of response relation and feature relation: performance is increased by feature repetition, only if the response repeats as well, but not if the response changes.

Results in many action control paradigms (e.g., repetition priming, negative priming, distractor–response binding, or response–response binding) can be explained as a result of such binding effects (Frings et al., 2020; Henson et al., 2014; Hommel et al., 2001; Moeller & Frings, 2019b). Binding and retrieval effects are typically found in the response time data and, somewhat less reliably, also in the error rates (e.g., Frings et al., 2007; Moeller & Frings, 2019b; for an exception see Mayr & Buchner, 2006). One factor proposed to influence binding effects and thus influencing whether an effect appears in the RTs, the error rates, or both is the current speed–accuracy settings of the participants (Frings et al., 2020). Different speed–accuracy settings can be induced by a multitude of factors, e.g. via deadlines, payoffs, or instructions, and can prompt a participant to trade accuracy for speed (or vice versa), a so-called speed–accuracy trade-off (Heitz, 2014; e.g., Wickelgren, 1977). These speed or accuracy settings lead to differences in RTs and error rates, with generally faster RTs but more errors under a speed setting and slower RTs but fewer errors under an accuracy setting (e.g., Fitts, 1966; Hale, 1969; Howell & Kreidler, 1963). This sort of variability in response choice can be accounted for by the diffusion model of Ratcliff (e.g., Ratcliff, 1978; Ratcliff & McKoon, 2023; Ratcliff & Rouder, 1998, 2000), proposing that information about stimuli and their identity is accumulated over time and only if a decision criterion is reached a decision is made, e.g. a response is given. Speed or accuracy settings serve to alter the decision criterion. A speed setting leads to a lower criterion, meaning that less evidence about stimuli and an appropriate response is needed for a decision, which leads to more errors, but faster decisions. On the other hand, an accuracy setting leads to a higher decision criterion, meaning that more evidence is accumulated before a decision is made, then resulting in fewer errors but also slower responses (Ratcliff & Rouder, 1998). Recently, it has been shown that the effect of different responding modes, trading speed for accuracy, also depend on the time course of the cognitive processes in effect in the current task (Heuer & Wühr, 2023; Mittelstädt et al., 2022). For example, the effect of distractor congruency information that unfolded later in time (in an Erikson flanker task) was diminished by a response mode maximizing speed, while an effect of distractor congruency that was encoded early on (in a Simon task), benefited from a speed instruction (Mittelstädt et al., 2022).

Notably, speed and accuracy are oftentimes mentioned as a standard in instructions of experiments. One intention here is to ensure responsible participation and thus maximize the probability to find the effect of interest. Regarding speed and accuracy, such instructions seem to aim for a criterion that excludes both very long response times and an abundance of errors: oftentimes both speed and accuracy are stressed, i.e. instructions are ambivalent, prompting participants to answer as quickly and as accurately as possible. Here, we aim to analyze whether this kind of instruction can also affect observed binding effects. To this end, we either stressed only accuracy or only speed in the instructions to a binding task and compared these conditions to a baseline condition that stressed both speed and accuracy.

The most obvious prediction is that we may find a simple speed–accuracy trade-off so that the binding effect is observable mostly in the response times under accuracy instructions and mostly in the error rates under the speed instructions, with generally no effect of the instructions on the magnitude of the binding effect (see e.g., Liesefeld & Janczyk, 2019). Yet, another possibility is that differences in speed and accuracy instructions modulate the magnitude of the measured binding effects. A change specifically in participants’ response criterion in the direction of less accuracy might entail a change in executive control, increasing the chance of influence due to additional mechanisms (e.g., distracting information, see Heitz & Engle, 2007). In turn, binding effects that are due to automatically triggered binding and retrieval processes might be more likely observed under speed than under accuracy conditions, leading to increased observed effects in a speed condition.

**Fig. 1:**
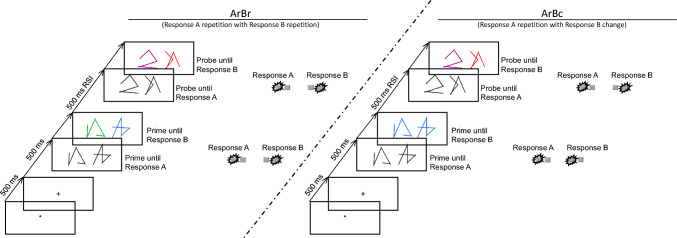
Sequence of events in two example trials of two different conditions in Experiments 1a and b. Note: Participants decided for each prime and each probe whether the presented stimuli had identical or different shapes (Response A) and identical or different colors (Response B). Left hand side: Example of a Response A repetition and Response B repetition trial (ArBr); Right hand side: Example of a Response A repetition and Response B cange trial (ArBc). The stimuli are not drawn to scale

To investigate the influence of instruction-induced speed and accuracy settings on binding effects, a speed vs. accuracy instruction manipulation was applied to a response-response (RR)-binding paradigm (Moeller & Frings, 2019b). RR-binding effects are typically investigated using trials with a prime–probe structure that includes two individually planned and executed responses both in the prime and in the probe (Moeller & Frings, 2019b; see Fig. 1).

It can be assumed that the consecutively given prime responses are bound upon execution. If one of the bound responses repeats as the first probe response, the other response is retrieved. If the retrieved response matches the required second probe response (Fig. 1, left hand side), retrieval facilitates response execution. If a different response is required as the second probe response (Fig. 1, right hand side), i.e. the retrieved and required responses are incompatible, retrieval leads to significantly less facilitation or even impairment, signified by higher error rates and longer response times. In accordance with the speed–accuracy trade-off literature, a speed vs. accuracy instruction manipulation should result in generally shorter RTs and higher error rates under speed instructions and likewise longer RTs but fewer errors under accuracy instructions. We furthermore expect that speed vs. accuracy instructions induce a shift in whether RR-binding effects are observed in RTs or error rates. In two online Experiments, we looked at a baseline condition of RR-binding with standard, ambivalent instructions (Experiment 1a) and set this in relation to conditions with speed vs. accuracy instructions (Experiment 1b). To anticipate results, instructions affected error rate binding effects, with the strongest binding effects under speed instructions, followed by ambivalent and then accuracy instructions. However, instructions had no impact on RTs and RT-binding effects.

## Experiment 1a

### Methods

#### Participants

The sample size was matched to those of past studies, investigating and finding response–response binding effects (Moeller & Frings, 2019a, 2019b, 2019c). Twenty-eight students (26 women) from Trier University participated in the experiment. The samples’ median age was 21.5 years, with a range from 18 to 38 years. The participants were rewarded with partial course credit. According to standard exclusion criteria of past binding studies, two additional participants had to be excluded, because they were far outs on error rates (more errors than three times the interquartile range above the third quartile of the sample’s error distribution).

#### Design

The design comprised two within-subjects factors, namely, response A relation (response repetition vs. response change from prime to probe), and response B relation (response repetition vs. response change from prime to probe).

#### Materials

The experiment was programmed in PsychoPy3/PsychoJS (2020.2.6; Peirce et al., 2019) and conducted online on Pavlovia (https://pavlovia.org/). For participation, a computer with a physical keyboard was required. Instructions were presented in white (RGB_255_: 255, 255, 255) on a grey background (RGB_255_: 128, 128, 128). The list of possible stimuli consisted of eight different shapes that were approximately 70 × 70 pixels in size and made up of four overlapping lines of different lengths. The shapes could be presented in eight different colors: blue (RGB_255_: 45, 120, 232), green (RGB_255_: 18, 186, 46), red (RGB_255_: 255, 0, 0), yellow (RGB_255_: 255, 252, 0), purple (RGB_255_: 164, 45, 232), brown (RGB_255_: 98, 58, 0), and orange (RGB_255_: 255, 144, 0). In each display, 2 shapes were presented simultaneously 65 pixels to the left and right of the screen center. Participants responded via four keys on a computer keyboard.

#### Procedure

Before the experiment, participants gave informed consent regarding the recording of personal data and responses during the experiment and indicated their age and gender. Instructions were given on the screen. Participants were instructed to place their middle and index fingers on the keys G, H, K, and L. They were told that they would always see two line patterns that would be either identical or different in shape and identical or different in color. Their task was always to first categorize the shapes (Response A) and then the colors (Response B) of these patterns as identical or different, by successively pressing two keys with the corresponding fingers. The left index and middle fingers were used for the shape classification. For identical shapes, participants were instructed to press the key with the left index finger (H) and for different shapes, they were supposed to press the key with the left middle finger (G). To classify the colors, the index and middle fingers of the right hand were used, respectively. For identical colors, a key was pressed with the right index finger (K), and for different colors, a key was pressed with the right middle finger (L).

An asterisk that was presented for 500 ms in the middle of the screen indicated the beginning of each trial (see Fig. 1). Then, a plus sign appeared for 500 ms, followed by the prime line patterns. These were presented in white for the shape comparison and, in the case of a correct response, changed color upon Response A execution (via the left hand). The colored shapes remained on the screen until Response B (via the right hand) was given. During training trials, a feedback message appeared on screen for 600 ms immediately following the response, indicating whether the given response was correct or not. Afterward, a blank screen appeared for 500 ms and was followed by the probe line patterns. The procedure in the probe was identical to that in the prime. Every 40 trials participants were allowed to take a short break, after which they resumed the task in their own time. In Response A repetition trials (Ar), the same response was required to the shapes of the prime and probe line patterns (e.g., the prime shapes differed, and the probe shapes differed). In Response A change trials (Ac), different responses were required for the categorization of the prime and probe line patterns (e.g., the prime shapes were identical, and the probe shapes differed). In Response B repetition trials (Br), the same response was required to the colors of the prime and probe line patterns (e.g., the prime colors were identical, and the probe colors were also identical). In Response B change trials (Bc), different responses were required to the prime and probe colors (e.g., the prime colors differed, and the probe colors were identical). These relations resulted in the four conditions Response A repetition with Response B repetition (ArBr), Response A repetition with Response B change (ArBc), Response A change with Response B repetition (AcBr), and Response A change with Response B change (AcBc). Each of these conditions was presented 8 times with each of the four possible combinations of identical/different shapes and colors in the probe, resulting in 128 experimental trials. Shapes and colors were randomly assigned to the different positions/displays. Before the experimental block started, participants first completed a training where participants practiced their task for at least 16 trials (subsample of the experimental trials). During the task instructions, the participants were told to respond as quickly as possible without making errors.

### Results

The dependent variable of interest was the performance in probe Response B. If prime Responses A and B are integrated, repeating prime Response A in the probe should trigger retrieval of the later response and thus influence performance on probe Response B. Only trials with correct responses A and B in both prime and probe were considered. The error rate for prime responses (A or B) was 9.4%. The probe error rates were 3.0% for Response A and 4.2% for Response B (only including trials with correct responses up to the reported response). We excluded RTs of more than 1.5 interquartile ranges above the third quartile of the probe Response B RT distribution of the individual participant (Tukey, 1977) and RTs shorter than 200 ms from the RT analysis. Due to these constraints, 20.1% of the trials were excluded from the RT analyses. For the error rate analyses, we included all trials that were correct in prime responses A and B and probe Response A, but incorrect in probe Response B, independent of response times. For the mean RTs and error rates, see Table 1.

**Table 1:** Mean response times (in milliseconds) and mean error rates (in percentages) for probe Response B, as a function of Response A relation and Response B relation and instruction (Experiment 1a: ambivalent; Experiment 1b: accuracy, speed)

	Ambivalent instruction		Accuracy instruction			Speed instruction	
	B repetition	B change	B repetition	B change		B repetition	B change
A change	678 (4.7)	643 (2.0)	662 (4.2)	646 (1.7)		578 (8.5)	567 (2.7)
A repetition	629 (3.1)	644 (6.9)	632 (4.2)	651 (3.0)		550 (5.2)	577 (9.0)
Priming	49 (1.6)	– 1 (– 4.9)	30 (0.0)	– 5 (– 1.3)		28 (3.3)	– 10 (– 6.3)
Binding Effect	50 (6.5)		35 (1.3)			38 (9.6)	

Priming of probe response B by repetition of response A from the prime (response A change minus response A repetition) is calculated for response B repetition and response B change conditions. The difference between these priming effects is the binding effect: Priming of response B is only beneficial if the primed response repeats. It impairs performance, if a different than the primed response is required

In a 2 (Response A relation: repetition vs. change) × 2 (Response B relation: repetition vs. change) analysis of variance (ANOVA) on probe Response B RTs, the main effect for Response A relation was significant, *F*(1, 28) = 37.41, *p* < 0.001, *η*_*p*_^*2*^ = 0.57, while the main effect for Response B relation was not, *F*(1, 28) = 3.35, *p* = 0.077, *η*_*p*_^*2*^ = 0.11. More importantly, the interaction of Response A and Response B relation was significant, *F*(1, 28) = 24.26, *p* < 0.001, *η*_*p*_^*2*^ = 0.46, indicating binding between the responses: the repetition of Response A facilitated performance only if Response B was repeated as well, *t*(28) = 7.23, *p* < 0.001, but not if Response B changed, *t*(28) = – 0.18, *p* = 0.859.

In the same analysis on error rates, the main effects of Response A relation, *F*(1, 28) = 3.19, *p* = 0.085, *η*_*p*_^*2*^ = 0.10, and Response B relation, *F*(1, 28) < 1, *p* = 0.628, *η*_*p*_^*2*^ = 0.01, were not significant. However, the interaction of Response A and Response B relation was significant, *F*(1, 28) = 13.37, *p* < 0.001, *η*_*p*_^*2*^ = 0.32, again indicating binding between the responses: The repetition of Response A numerically facilitated performance if Response B was repeated as well, *t*(28) = 1.87, *p* = 0.071, but impaired performance if Response B changed, *t*(28) = 4.13, *p* < 0.001.

### Discussion

In line with existing literature, we found significant RR-binding effects in both, RTs and error rates under ambivalent instructions obtained in an online setting. With this as a baseline, Experiment 1b set out to investigate whether RR-binding is affected by instructed speed vs. accuracy settings. If instructions induce speed and accuracy settings, this should influence mean RTs and error rates, with comparably faster RTs, but more errors under speed instructions than accuracy instructions, in line with findings on speed-accuracy instruction manipulations (e.g., Hale, 1969; Howell & Kreidler, 1963). Furthermore, we expect speed vs. accuracy instructions to induce a shift in whether RR-binding effects are observed in RTs or error rates.

## Experiment 1b

### Methods

#### Participants

Again, the sample size was approximated to those of past studies investigating response–response binding. Twenty-eight students (18 women) from Trier University participated in the online experiment. The samples’ median age was 23 years, with a range from 19 to 56 years. The participants were rewarded with partial course credit.

#### Design

The design comprised two within-subjects factors, namely, response A relation (response repetition vs. response change from prime to probe), and response B relation (response repetition vs. response change from prime to probe), and one between-subject factor, instructions (accuracy vs. speed instruction).

#### Materials and procedure

Materials and procedure were identical to Experiment 1a with the following exceptions. Each of the 4 possible Response A and B repetition and change conditions (ArBr, ArBc, AcBr, AcBc) was presented 12 times, resulting in 192 experimental trials. Before the experimental block started, participants first completed a short pre-training explaining the task, followed by a training where participants practiced their task for at least 16 trials (subsample of the experimental trials) and had to pass a 75% accuracy threshold to proceed to the main experiment. This practice was added to ensure participants understood and followed the task rules. Depending on the condition, an accuracy vs. speed manipulation was implemented: during the task instructions, the participants were told to answer either as fast as possible (speed) or as correctly as possible (accuracy). Additionally, participants received condition-dependent feedback on mean response speed (in ms; in the speed instruction condition) or mean accuracy (in %; in the accuracy instruction condition) every 12 trials. This feedback was meant to ensure that participants kept the instructed speed or accuracy response mode active throughout the experiment.

### Results

As in Experiment 1a, only trials with correct responses A and B in both prime and probe were considered. The error rate for prime responses (A or B) was 8.0%. The probe error rates were 2.8% for Response A and 4.5% for Response B (only including trials with correct previous responses). We excluded RTs of more than 1.5 interquartile ranges above the third quartile of the probe Response B RT distribution of the participant (Tukey, 1977) and RTs shorter than 200 ms from the analysis. Due to these constraints, 18.1% of the trials were excluded from the RT analyses. For the mean RTs and error rates, see Table 1.

The dependent variable of interest was again performance in probe Response B. In a 2 (Response A relation: repetition vs. change) × 2 (Response B relation: repetition vs. change) × 2 (task instructions: accuracy vs. speed) ANOVA on probe Response B RTs the main effect for instructions was not significant, *F*(1, 26) = 2.06, *p* = 0.163, *η*_*p*_^*2*^ = 0.07, indicating that the instruction manipulation had no impact on RTs. The main effect for Response A relation was significant, *F*(1, 26) = 5.43, *p* = 0.028, *η*_*p*_^*2*^ = 0.17, while the main effect for Response B relation was not, *F*(1, 26) < 1, *p* = 0.452, *η*_*p*_^*2*^ = 0.02. More importantly, the interaction of Response A and Response B relation was significant, *F*(1, 26) = 19.41, *p* < 0.001, *η*_*p*_^*2*^ = 0.43, indicating binding between the responses. However, this was not further modulated by task instructions, *F*(1, 26) < 1, *p* = 0.895, *η*_*p*_^*2*^ < 0.01. RT-binding effects were significantly different from zero for both, speed instructions, *t*(12) = 3.11, *p* = 0.009, and accuracy instructions, *t*(14) = 3.12, *p* = 0.007.

**Fig. 2:**
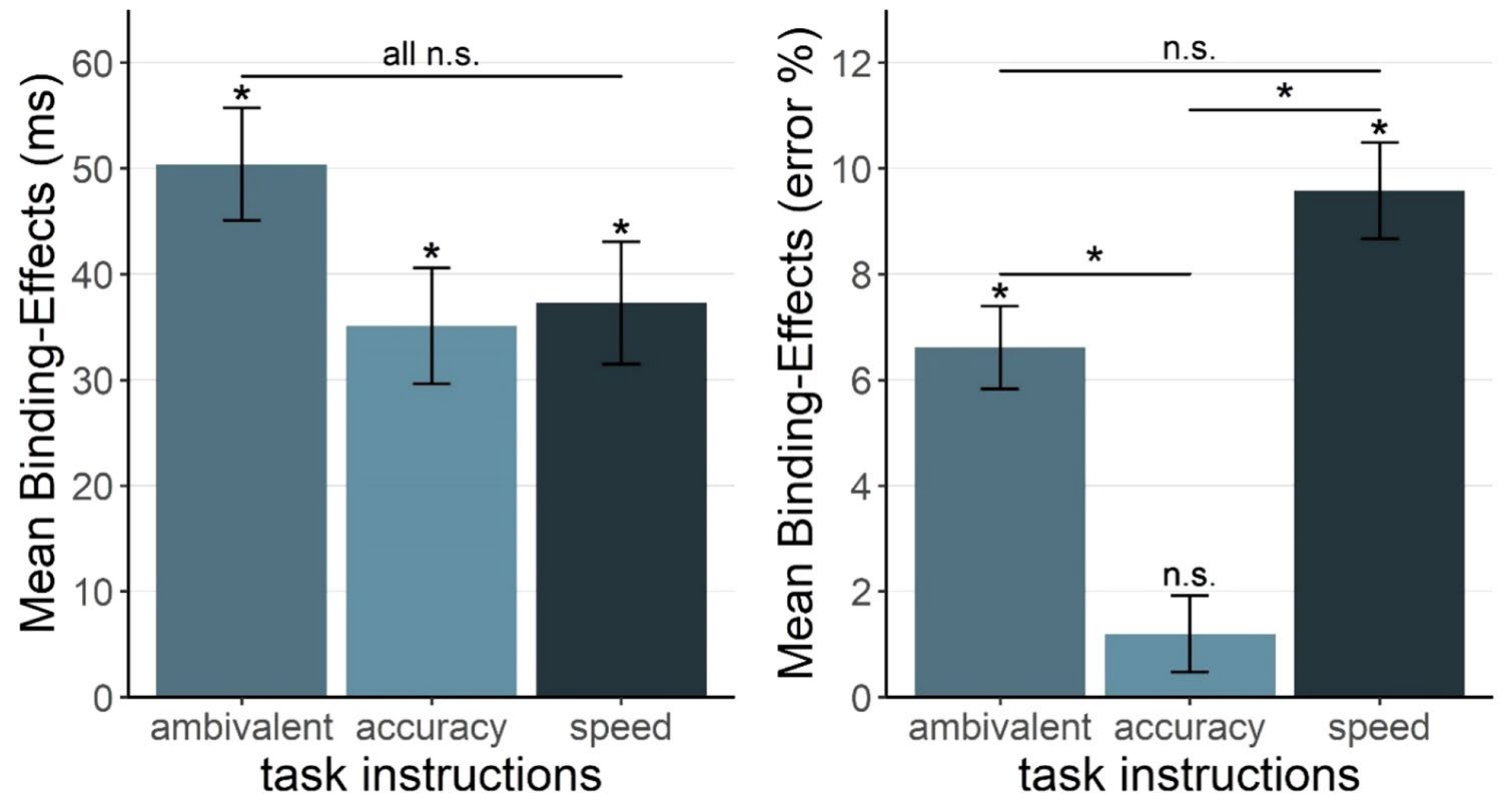
Mean response-response binding effects for response times and error rates in Experiments 1a and 1b as a function of task instructions (accuracy vs. ambivalent vs. speed). Note. Binding effects are calculated as the advantage of probe Response A repetition (vs. probe Response A change; i.e., priming effect, see Table 1) in probe Response B repetition trials minus the advantage of probe Response A repetition (vs. probe Response A change) in probe Response B change trials: [AcBr–ArBr]–[AcBc–ArBc]

In the same analysis on error rates, the main effect of task instructions was significant, *F*(1, 26) = 7.20, *p* = 0.012, *η*_*p*_^*2*^ = 0.22, signifying an influence of task instructions on error rates. There were higher mean error rates in the speed condition (*M* = 6.32%) than in the accuracy condition (*M* = 3.27%). The main effect of Response A relation, *F*(1, 26) = 4.85, *p* = 0.037, *η*_*p*_^*2*^ = 0.16 was again significant, while the main effect of Response B relation, *F*(1, 26) = 2.94, *p* = 0.098, *η*_*p*_^*2*^ = 0.10, was not. The interaction of Response A and Response B relation was significant, *F*(1, 26) = 18.63, *p* < 0.001, *η*_*p*_^*2*^ = 0.42, again indicating binding between the responses. Importantly, this relation was further modulated by task instructions, *F*(1, 26) = 12.54, *p* = 0.002, *η*_*p*_^*2*^ = 0.33. Under speed instructions, a significant error rate binding effect emerged, *t*(12) = 5.10, *p* < 0.001, while it was not significant under accuracy instructions, *t*(14) = 0.81, *p* = 0.431. In sum, results suggest a modulating influence of task instructions on RR-binding only for error rates. For a summary of mean binding effects, see Fig. 2.

#### Comparison of experiments 1a and 1b

Additionally, we compared results from the instruction manipulation of Experiment 1b with the results obtained under ambivalent instructions in Experiment 1a. In a 2 (Response A relation: repetition vs. change) × 2 (Response B relation: repetition vs. change) × 3 (task instructions: accuracy vs. ambivalent vs. speed) ANOVA on probe Response B RTs, again the main effect for task instruction was not significant, *F*(1, 53) = 2.34, *p* = 0.106, *η*_*p*_^*2*^ = 0.08. The main effect for Response A relation was significant, *F*(1, 53) = 32.58, *p* < 0.001, *η*_*p*_^*2*^ = 0.38, while the main effect for Response B relation was not, *F*(1, 53) < 1, *p* = 0.521, *η*_*p*_^*2*^ = 0.01. The interaction of Response A and Response B relation was significant, *F*(1, 53) = 40.64, *p* < 0.001, *η*_*p*_^*2*^ = 0.43, indicating binding between responses. This was again not further modulated by task instructions, *F*(2, 53) < 1, *p* = 0.574, *η*_*p*_^*2*^ = 0.02.

The same analysis on error rates revealed a significant main effect for task instructions, *F*(2, 53) = 3.84, *p* = 0.028, *η*_*p*_^*2*^ = 0.13, again indicating an influence of instructions on error rates. The main effect was significant for Response A relation, *F*(1, 53) = 8.10, *p* = 0.006, *η*_*p*_^*2*^ = 0.13, but not for Response B relation, *F*(1, 53) = 0.41, *p* = 0.525, *η*_*p*_^*2*^ = 0.01. Again, the interaction of Response A and Response B relation was significant, *F*(1, 53) = 34.84, *p* < 0.001, *η*_*p*_^*2*^ = 0.40. Importantly, this relation was modulated by task instructions, *F*(2, 53) = 4.73, *p* = 0.013, *η*_*p*_^*2*^ = 0.15. Post hoc t-tests (holm corrected, Holm, 1979) revealed significant differences between error rate binding effects in the speed and accuracy instruction conditions, *t*(23.74) = 3.50, *p* = 0.006, and between ambivalent and accuracy instructions, *t*(38.34) = 2.49, *p* = 0.034, while the difference between speed and ambivalent instructions was not significant, *t*(28.76) = 1.20, *p* = 0.238. For a summary of binding effects, see Fig. 2.^1^

### Discussion

Results from Experiment 1b show that the instruction manipulation affected mean error rates, but had no general impact on mean RTs, as signified by the respective main effects. This is in line with previous findings, where instructions seemed to have a stronger impact on response accuracy, while response speed was less affected (Howell & Kreidler, 1963). Consequently, instructions did also not influence binding effects in RTs. However, the speed vs. accuracy instruction manipulation affected error rate binding effects, with significantly stronger RR-binding effects under speed than under accuracy instructions. Additionally, ambivalent instructions of Experiment 1a functioned as a middle category, with both, medium error rates and error rate binding effects compared to the other two instruction conditions.

## General discussion

In this study, we investigated the influence of instruction-induced speed and accuracy settings on binding effects by varying instructions to participants, working through an RR-binding task. Instructions modulated error rates and error rate binding effects but did not influence results in RTs. Participants seem to have adjusted their accuracy criterion according to the instruction, while they did not alter their response speed. Apparently, a simple instruction focusing on speed or accuracy, respectively, is not sufficient to tip the speed-accuracy trade-off in one or the other direction. In fact, there might be other prevailing influences like the personal motivation of the participant to be quick or accurate in their responses or the expectations evoked by the experimental setting. For example, in the present experimental setup, each response terminated the presentation of a given stimulus. That is, fast responding would decrease the time spent, participating in the experiment. It is possible that this led to a general motivation for fast responding so that an additional speed instruction could not influence reaction times much more. In the present study, this may have led to the effect of instruction being smaller on response times than on error rates. Together with a relatively limited sample size, the power was likely not sufficient for the difference in response times to lead to a significant result. Gaining a deeper understanding of these kinds of personal motivations, including possible individual differences in response mode adjustment under different instructions, could be an interesting line of research on its own.

Even though we did not find evidence for a classical speed-accuracy trade-off, instructions did affect performance. Error rate analyses indicated both, more errors and larger binding effects in the speed condition. The highest error rate binding effects occurred under speed instructions, followed by ambivalent instructions, while there were no significant binding effects under accuracy instructions. Additionally, error rate binding effects under accuracy instructions differed significantly from the other two instruction conditions. To explain these results, we can only speculate that participants interpreted the speed instructions as not needing to worry about accuracy rather than focusing on speed. In turn, they did not increase speed, which is apparent in our RT results, but only lowered their effort. This might have resulted in reduced executive control and slower focus of attention, in turn leading to more influence of potentially distracting information like the retrieved response (see Heitz & Engle, 2007) and thus in larger error rate binding effects under speed instructions. If this reasoning is correct, we may assume that speed instructions would generally allow for more influence of automatic processes in responding.

The diminished error rate binding effects under accuracy instructions compared to speed or ambivalent instructions indicate that binding effects seem observable in the error rates only when participants (at least partially) focus on speed. For most research questions, it might not be relevant whether (binding) effects occur in RTs or error rates, but knowing about the influence of different instructions helps to set expectations on where to find effects accordingly. Thus, if one is interested to find error rate effects, speed-focused, or at least ambivalent instructions should be considered. On the other hand, regarding RTs, the choice of instructions appears to be less impactful—at least in this online setting—as binding effects were observed either way. From this, we can derive two things: first, the typical focus in previous studies on RTs as the main dependent variable of interest seems to be sensible. Second, if there is no theoretical or practical reason to focus only on accuracy in the instructions, mentioning speed in addition seems to be generally advisable, as chances seem to be higher to observe effects in both dependent variables. Further, this finding is likely generalizable to a number of related effects. For example, response priming effects, task switching effects, negative priming effects, sequential compatibility effects, and effects of action planning, have been shown to rely partly on the same mechanisms responsible for binding effects in the present study (see Frings et al., 2020; Henson et al., 2014). Therefore, the binding and retrieval mechanisms in these paradigms are likely affected by speed and accuracy instruction in a similar way.

Our results are in line with the finding that the effect of distractor information can be increased by a speed instruction, only if the relevant distractor compatibility information is processed early on (Heuer & Wühr, 2023; Mittelstädt, et al., 2022). With the retrieving information being a response and thus central to the task, we can assume early processing of the retrieved information, in the present study. Taken together, it is thus possible that the current findings will mainly generalize to effects that rely on retrieval of features, central to the task (e.g., target- or response features). Whether the pattern of influence is more complicated if retrieval is triggered by the repetition of a task irrelevant feature remains an open issue.

Note that the result pattern in the ambivalent condition was more similar to the speed condition, with binding effects significant in both RTs and error rates and also not significantly different from each other. One possible interpretation would be that the less formal online setting of the study led to a general motivation to pass the experiment fast, and critically that not much effort for accuracy was necessary. This pattern of results is also in line with previous research on binding effects, indicating that binding effects in general do not differ significantly between online and offline settings, but that there is a tendency for stronger error rate binding effects online (Moeller & Frings, 2021). That is, an online setting might lead to a tendency to deprioritize accuracy. Fortunately, it seems that such a shift in operation mode, if anything, facilitates the measurement of these effects.

Our results fit in with the Binding and Retrieval in Action Control framework (Frings et al., 2020), which proposes that binding and retrieval processes can be modulated by different bottom-up and top-down influences and specifically that top-down influences can act on different representational levels, for example, mindsets, speed–accuracy tradeoffs, or instruction-based effects. Our study provided evidence that instructed speed–accuracy settings do indeed modulate whether binding and retrieval processes affect overt behavior. This finding might be explained by an altered amount of executive control under the different instruction conditions. Executive control was previously found to be important for the retrieval process, in that factors associated with less efficient executive control (e.g., lower scores on fluid intelligence measures or autism spectrum disorder) are also associated with more partial repetition costs (Colzato et al., 2006; Zmigrod et al., 2013; for an overview, see Hommel, 2022), thus resulting in stronger binding effects. This is consistent with the present results: when RTs and error rates are considered together, we find stronger overall binding effects for speed instructions, i.e., instructions that we hypothesize exert the least amount of executive control. Even though we cannot distinguish with this type of modulation to what extent binding and retrieval processes were independently affected by the instructions, the broad agreement in the literature seems to be that the retrieval process is generally more easily affected by modulations than the binding process (Hommel, 2022; Hommel et al., 2014; Moeller & Frings, 2014). This, together with the previously found influences of executive control on retrieval, suggests that also in the present data pattern it was most likely the retrieval process that was affected by the instruction modulation.

In sum, the results at hand provide evidence that instructed speed and accuracy can affect observed binding effects. A focus on speed rather than accuracy in the instructions resulted in larger binding effects in error rates, while observed binding effects in RTs remained largely unaffected by instructions. Hence, on a practical note, for effects regarding automatic processes (like binding and retrieval) to show up in a data set, it might be reasonable to focus more on speed than on accuracy in the instructions.

### Supplementary Information

Below is the link to the electronic supplementary material.

Supplementary file1 (DOCX 14 KB)

## Data Availability

The data and analysis code of the current study are available in the Psycharchives repository under 10.23668/psycharchives.7967 (data) and 10.23668/psycharchives.7966 (code).

## Footnotes

1 Since the data for Experiment 1a and Experiment 1b were collected separately, not randomly assigning participants to the different experiments, we conducted a control analysis including the factor experiment to be sure that the factor experiment did not generally affect response times or binding effects. In a 2 (Response A relation) × 2 (Response B relation) × 2 (Experiment) ANOVA on probe Response B RTs, the main effect of experiment was not significant, *F*(1, 54) = 1.41, *p* = 0.241, *η*_p_^2^ = 0.03, and the factor experiment did not modulate the binding effect significantly, *F*(1, 54) = 1.13, *p* = 0.293, *η*_p_^2^ = 0.02. In the same analysis on error rates, again the main effect of experiment, *F*(1, 54) < 1, *p* = 0.540, *η*_p_^2^ = 0.01, and the interaction of experiment with the binding effect, *F*(1, 54) < 1, *p* = 0.476, *η*_p_^2^ = 0.01, did not reach significance.
