# Supplementary material for: Instructed speed and accuracy affect binding
Source: Psychol Res. 2024 Feb 9;88(4):1203–11. doi: 10.1007/s00426-024-01927-y (PMC11142972; doi:10.1007/s00426-024-01927-y)
Supplement: Supplementary file 1 — Supplementary file1 (DOCX 14 KB) [file 426_2024_1927_MOESM1_ESM.docx]

***Supplementary Information***

**Table A.** *Mean response times (RT) in ms and mean error rates (ER) in % of prime Response A, prime Response B, and probe Response A in the conditions ambivalent, speed, and accuracy instructions*

|  | Probe Response A | |  |  | Prime Response B | |  |  | Prime Response A | |  |
| --- | --- | --- | --- | --- | --- | --- | --- | --- | --- | --- | --- |
|  | ambivalent | accuracy | speed |  | ambivalent | accuracy | speed |  | ambivalent | accuracy | speed |
| mean RT | 915 | 839 | 805 |  | 692 | 680 | 587 |  | 897 | 846 | 786 |
|  |  |  |  |  |  |  |  |  |  |  |  |
| mean ER | 2.54 | 3.02 | 4.01 |  | 3.99 | 4.69 | 6.21 |  | 2.93 | 3.37 | 3.87 |
